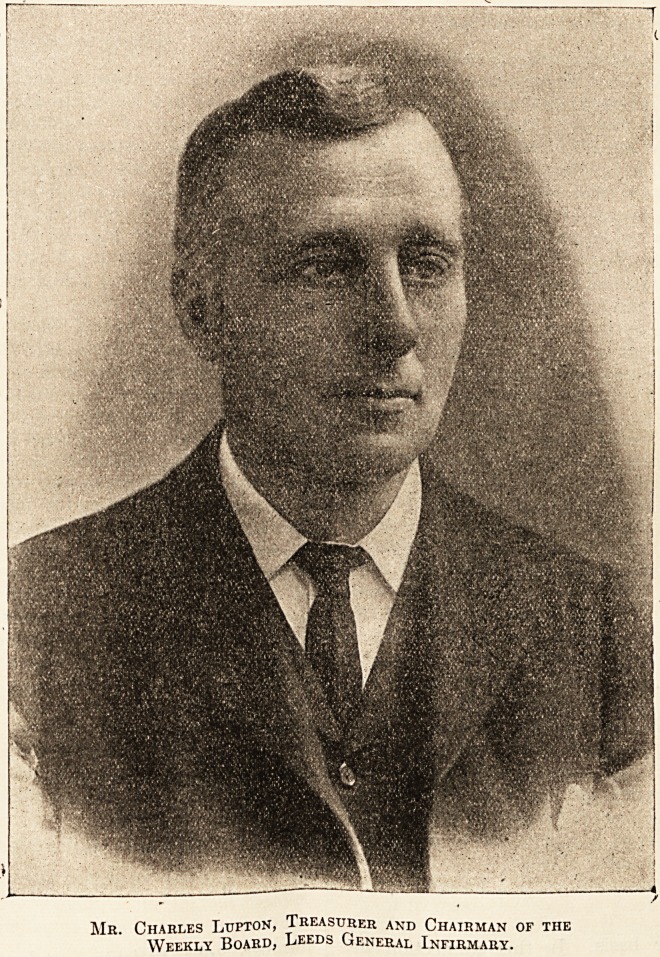# Eminent Chairman Series

**Published:** 1911-02-11

**Authors:** 


### SPECIAL INSTITUTIONAL ARTICLE.

### EMINENT CHAIRMAN SERIES.

### V.—MR. CHARLES LUPTON,

### CHAIRMAN AND TREASURER OF THE LEEDS GENERAL INFIRMARY.

For some eleven years Mr. Charles Lupton has ably discharged the responsible duties of Treasurer and Chairman of the Weekly Board of the Leeds Infirmary. Mr. Lupton is by inheritance, training, and disposition not only an able but a most capable administrator. His procedure seems to be based upon the wise belief that it is essential to success that every responsible administrator shall first ascertain for himself in all its details the requirements of every department before countenance is given to demands for alterations or extensions. Holding these views, Mr. Lupton has gradually obtained the fullest possible knowledge of the interior working, in all its departments, of the hospital he controls, and he is entitled to-day to be regarded as one of the best informed and knowledgeable of hospital managers.

The report which we gave in The Hospital of January 29, 1910, of the institution which Mr. Lupton controls, demonstrates the courage and resolution which he displayed when he accepted office in 1900. At that time the Leeds General Infirmary had arrived at a critical period in its history. The expenditure exceeded the income by upwards of £8,000, and most of the departments and the whole of the buildings required to be taken in hand with a view to their being made, in the modern hospital sense, worthy of a great city like Leeds. Mr. Lupton's difficulties were and have been immensely increased by the form of construction adopted when the present buildings were erected in 1864. It is not necessary to repeat here what we have said on this point in the article to which reference has already been made. It was not possible for the new Treasurer to attempt to set his house in order from top to bottom at the outset. The first requisite to success was to think out carefully all the problems presented and then to take up the work piece by piece, year by year, as events and circumstances permitted.

Mr. Charles Lupton, Treasurer and Chairman of the Weekly Board, Leeds General Infirmary.

Mr. Lupton found that the original objects of the founders of the Leeds Infirmary in 1768 had been allowed to be seriously departed from. The founders declared their design to be to supply the honest and industrious poor, and all who under sickness or casualties are unable to supply themselves with such requisites, with adequate advice, medicine and other necessary means of cure. There are many useful and industrious working men and labourers, who whilst they are in health are able to provide for the present subsistence of themselves and their families, who cannot, with all their economy, make adequate provision against the time of sickness. Such worthy citizens, and a number of less thrifty folk, when overtaken by sickness or accident—having to cease work in consequence—find themselves unable to defray the cost of medical assistance. These are the cases to relieve whom the Leeds General Infirmary was originally founded in 1767. Like most of the great voluntary hospitals, as time has passed, and especially during the last thirty years when the popularity of voluntary hospitals, owing to the excellence of their management, has attracted all classes to their doors in the hope of obtaining treatment, abuses have crept in. Hence there is too much reason to fear that other classes than those which the founders had in mind, had, before Mr. Lupton's day, obtained free relief where, if strict justice had been done, they should have been called upon to defray the whole or a portion of its cost.

### Coping with Hospital Abuse.

Mr. Lupton, therefore, set to work to try to make the benefits of this great hospital largely revert to the classes the needs of which it was founded to supply. He succeeded first of all in narrowing the two main channels of abuse, *i.e.*, through the casualty department and the so-called ticket or note cases. He then took in hand the removal of these abuses, and at the present time treatment is refused to the servants of those who are able to afford to pay for it, and the idea that a subscriber is entitled to get value for his money, if not now entirely eradicated, has been put upon a higher and better basis. In this connection he has taken steps to secure the appointment of almoners, whereby every care is taken to bring to the notice of each patient that the institution exists for the free relief of those who are well able to provide for themselves and their families when well, but who cannot adequately defray the cost of sickness or accident. He has driven home the realisation that the Leeds Infirmary is not intended for those who can afford to pay on the one hand, nor for pauper cases who are adequately provided for and can be efficiently treated in one of the Poor-law infirmaries.

Again, Mr. Lupton has made himself master of the details of hospital administration, and has made it his business to prevent waste and to cut down unnecessary expenditure in every department. He has so far succeeded that a close inspection of the interior working proves him to be one of the most economical and careful managers to be met with amongst British hospital officials. So thorough is the control exercised over expenditure that we formed the conviction that, on the whole, if the present management errs at all, it errs on the side of economy. Perhaps this point has struck Mr. Lupton himself, for we observe that, whereas the cost of each out-patient remains about the same year by year, the cost of each in-patient has increased by 10s. a head. To succeed as a hospital administrator, it is essential that the treasurer should carry with him the co-operation and support of the medical and general staff. Recognising this, Mr. Lupton, when taking in hand the question of expenditure, caused a statement to be sent weekly to each member of the honorary medical staff, which contained the number of patients under his care, the amount of stimulants ordered, the cost of massage, and the extra nurses employed. Once a month each member of the honorary staff receives a statement of the cost of each of his patients for dressings and other items well within the control of the practitioner, whilst every sister and resident medical officer receives a statement of the expenditure of each ward and department of the Infirmary, so that they may realise how far the section of the work entrusted to them compares favourably or otherwise, in an economical sense, with that of each of their colleagues. By this means material reductions have been secured, and the average of expenditure in all departments and under all heads has been kept wonderfully uniform. Accounts are a necessary feature of hospital management, but to be efficient they must be kept upon an intelligent system. It is greatly to Mr. Lupton's credit that since he has been Treasurer the Infirmary has adopted the Uniform System of Accounts, so as to facilitate the comparison of the expenditure of the Leeds Infirmary upon an identical basis with other great hospitals throughout the country.

### Sources of Income.

In dealing with the question of income, Mr. Lupton seems to have examined into every source from which revenue comes. We trace his hand in the reorganisation of the system under which the Hospital Sunday Fund at Leeds has been apportioned. The new basis of awards assigns 70 per cent. to the Leeds General Infirmary; 14 per cent. to the Leeds Dispensary; 10 per cent. to the Hospital for Women and Children; 5 per cent. to the District Nursing Association, and 1 per cent. to the Leeds Maternity Home. In recent years the working classes have taken a much keener interest in the support of the Infirmary; the contributions derived from the workpeople show a steady increase, whilst annual subscriptions and collections in places of worship unfortunately exhibit a tendency to decrease. This latter decrease is not worthy of the citizens of a great city like Leeds. We commend this fact to their close attention in the hope that every thinking man and woman amongst them will make it his business to do his best to redress this falling off in charity at the very time when the managers have displayed an ever-increasing desire to secure economy with efficiency. Mr. Lupton has made it his business to raise by special appeal a considerable sum, with the result that he has succeeded in wiping out the heavy burden with which the Infirmary was handicapped when he took office. Speaking generally, it is fair to say that probably few great hospitals in this country have been more thoroughly or advantageously overhauled economically and administratively than the institutions which Mr. Charles Lupton has so ably administered for upwards of ten years.

### Modernising the Hospital.

But apart from the ordinary working of his hospital and the enforcement of its original objects, Mr. Lupton has displayed great energy and knowledge in providing for the introduction of modern appliances and for testing the possibility of remodelling old wards and making them efficient as modern units. He has ever displayed the greatest desire to place the medical staff in a position adequately to treat disease in each department. By installing the electric light, introducing and extending the Finsen Light Department, the provision of the high-frequency apparatus, the extension of the clinical laboratories, and by additions to the honorary medical staff, he has shown much commendable activity in these directions. Another great change whereby the average stay of each in-patient has been reduced from 28 to 18 days under the present treasurer has been the institution of two semi-convalescent hospitals outside Leeds, where patients recovering from operation or disease can be transferred and treated until recovery. These semi-convalescent hospitals have proved most successful. They are managed, and well managed, as hospitals, and they have considerably added to the bed accommodation of the General Infirmary.

### The Reconstruction Schemes.

Mr. Lupton has not hesitated to tackle the great question of reconstruction. During the last few years he has taken in hand several important internal structural alterations. A new ward of 32 beds has been opened. Some of the old wards have been refitted and refurnished; several of the floors have been relaid; the walls have been re-plastered, and the sanitary and kitchen arrangements of each ward have been thoroughly reconstructed and modernised. In this way by careful experiment and progressive care the committee have been enabled to claim, in the appeal they are now making for £150,000, that under their plan of reconstruction each of the old wards can be made as good as any modern ward. The work of reconstruction has been well thought out and represents an honest attempt, by remodelling and additions, to make the existing buildings up-to-date and efficient in the modern sense. We have already given an account of the works which this reconstruction will involve, and it is a striking testimony to the success attained by Mr. Charles Lupton, and to the great confidence which the people of Leeds repose in himself and his co-managers, that the appeal for £150,000, which was only decided upon at a public meeting held on January 26, has already, before the issue of any general appeal, produced half or nearly half the total sum required. If the working classes wish to have a sound reason why the voluntary system of hospital support has produced, on the whole, the most efficient hospitals in the world, we would point to the self-denying and unselfish devotion of the best of the great hospital managers who control these institutions. These great voluntary workers, of whom Mr. Charles Lupton is one, have set themselves whole-heartedly to understand the nature of the work and its responsibilities from every point of view. This accomplished, Mr. Lupton spared neither time nor energy in the attempt to make this work as good, as efficient and as fruitful in benefit to the suffering poor as has been humanly possible.

We hope the citizens of Leeds will testify their appreciation of the results we have here recorded, and their pride in their city and its institutions, by providing the whole sum of £150,000 required before the summer holidays, if not even by March 31, 1911, which latter would constitute a record.

## Figures and Tables

**Figure f1:**